# Targeting transforming growth factor-β signaling for enhanced cancer chemotherapy

**DOI:** 10.7150/thno.51383

**Published:** 2021-01-01

**Authors:** Jitang Chen, Ze-yang Ding, Si Li, Sha Liu, Chen Xiao, Zifu Li, Bi-xiang Zhang, Xiao-ping Chen, Xiangliang Yang

**Affiliations:** 1 National Engineering Research Center for Nanomedicine, College of Life Science and Technology, Huazhong University of Science and Technology, Wuhan, 430074, China.; 2 Hepatic Surgery Center, and Hubei Key Laboratory of Hepatic-Biliary-Pancreatic Diseases, National Medical Center for Major Public Health Events, Tongji Hospital, Tongji Medical College, Huazhong University of Science and Technology, Wuhan, China.; 3 Key Laboratory of Molecular Biophysics of Ministry of Education, College of Life Science and Technology, Huazhong University of Science and Technology, Wuhan, 430074, China.; 4 Hubei Key Laboratory of Bioinorganic Chemistry and Materia Medical, Huazhong University of Science and Technology, Wuhan, 430074, China.; 5 GBA Research Innovation Institute for Nanotechnology, Guangdong, 510530, China.

**Keywords:** transforming growth factor-β (TGFβ), extracellular matrix (ECM), tumor vasculature, epithelial-mesenchymal transition (EMT), cancer stem-like cells (CSCs), cancer chemotherapy

## Abstract

During the past decades, drugs targeting transforming growth factor-β (TGFβ) signaling have received tremendous attention for late-stage cancer treatment since TGFβ signaling has been recognized as a prime driver for tumor progression and metastasis. Nonetheless, in healthy and pre-malignant tissues, TGFβ functions as a potent tumor suppressor. Furthermore, TGFβ signaling plays a key role in normal development and homeostasis by regulating cell proliferation, differentiation, migration, apoptosis, and immune evasion, and by suppressing tumor-associated inflammation. Therefore, targeting TGFβ signaling for cancer therapy is challenging. Recently, we and others showed that blocking TGFβ signaling increased chemotherapy efficacy, particularly for nanomedicines. In this review, we briefly introduce the TGFβ signaling pathway, and the multifaceted functions of TGFβ signaling in cancer, including regulating the tumor microenvironment (TME) and the behavior of cancer cells. We also summarize TGFβ targeting agents. Then, we highlight TGFβ inhibition strategies to restore the extracellular matrix (ECM), regulate the tumor vasculature, reverse epithelial-mesenchymal transition (EMT), and impair the stemness of cancer stem-like cells (CSCs) to enhance cancer chemotherapy efficacy. Finally, the current challenges and future opportunities in targeting TGFβ signaling for cancer therapy are discussed.

## Introduction

Transforming growth factor-β (TGFβ) is a multifunctional cytokine that regulates numerous critical physiological functions in development, homeostasis, tissue regeneration, and immune tolerance 1-3. In particular, TGFβ signaling plays a dual role in cancer. During tumor initiation and early cancer stages, TGFβ suppresses tumorigenesis by inducing apoptosis of pre-malignant cells and inhibiting proliferation of cancer cells. However, in late-stage cancers, the tumor suppressor function of TGFβ is decreased through dysregulation of gene expression. TGFβ is overexpressed and becomes a main driver for tumor progression and metastasis 2, 4, 5. TGFβ induces epithelial‑to‑mesenchymal transition (EMT), which increases metastatic potential, drug resistance, and cancer cell stemness 6-10. TGFβ stimulates fibroblast proliferation and their transition to myofibroblasts or cancer-associated fibroblasts (CAFs), which overproduce extracellular matrix (ECM) components and exert physical forces to stiffen the ECM. The stiff, tumor-associated ECM, composed of collagen, hyaluronan, fibrin, and fibronectin, compresses blood and lymphatic vessels and increases the solid stress, leading to reduced tumor perfusion and oxygen delivery 11. Further, TGFβ regulates tumor angiogenesis and contributes to the formation of aberrant tumor vasculature, thereby interfering with the delivery of chemotherapeutic agents 12. TGFβ signaling impacts multiple immune cells, including macrophages, neutrophils, T cells, natural killer (NK) cells, dendritic and B cells, thus creating an immunosuppressive tumor microenvironment. For instance, TGFβ induces macrophages to polarize towards an M2 phenotype, thereby promoting an immunosuppressive microenvironment 13, 14. Similarly, TGFβ induces an N2 neutrophil phenotype, which promotes tumor progression and metastasis 15. In addition, TGFβ overexpression accelerates the formation of an immunosuppressive microenvironment by promoting the transformation of naïve T cells to regulatory T (Treg) cells 16, 17. TGFβ also suppresses the maturation of helper T (Th) cells 18, dendritic cells and NK cells 19-21. Furthermore, TGFβ can induce apoptosis of B cells, thereby further exacerbating immunosuppression 22. The effect of TGFβ on the tumor immune microenvironment and cancer immunotherapy has been reviewed in detail elsewhere 23-25. Therefore, blocking TGFβ signaling has significant clinical potential for treating late-stage cancers and tumor metastasis 11. However, TGFβ inhibition alone could facilitate tumor growth and metastasis. For example, inhibition of TGFβ decreases ECM deposition, alleviates physical forces, decompresses blood vessels, and improves blood perfusion. Increased blood perfusion could increase the supply of nutrients and oxygen to cancer cells, leading to enhanced tumor growth 11. Furthermore, reduced tumor ECM deposition could interfere with cell-cell or cell-stroma junction formation 26. This could increase the potential of metastatic cancer cells to escape the primary tumor via decompressed vessels and ECM 27, 28. Hence, targeting TGFβ signaling alone for cancer therapy is still controversial. Given the multifaceted roles of TGFβ signaling in maintaining physiological homeostasis, off-target toxicity is a major hurdle for the clinical translation of TGFβ blocking agents 29, 30.

TGFβ signaling severely hinders the clinical efficacy of chemotherapy, which is still the gold standard for cancer treatment and can increase the overall survival (OS) of cancer patients. However, traditional chemotherapeutic agents are not specific for tumor cells, leading to serious side effects and reduced efficacy. Although different nanomedicine approaches have been developed to reduce chemotherapy side effects and to improve efficacy through precise drug delivery to tumor tissues, it is difficult to eradicate malignant cells with chemotherapy alone. In advanced solid tumors, TGFβ overexpression can reduce chemotherapy efficacy by (1) excessive ECM deposition (collagen, hyaluronan, fibrin), which creates a dense physical barrier hampering the penetration of chemotherapeutic drugs into the tumor; (2) aberrant blood vasculature interfering with drug delivery; (3) EMT, which is accelerated by chemotherapeutic agents, leading to carcinoma cell dissemination and drug resistance; and (4) cancer stem-like cells (CSCs, or tumor initiating cells; TICs) resistant to chemotherapeutic drugs causing tumor relapse 31. Therefore, TGFβ signaling plays a central role in creating an aberrant TME, thereby limiting chemotherapeutic drug delivery and antitumor efficacy. Furthermore, heterogeneous drug distribution in the TME promotes TGFβ secretion, which further limits chemotherapy efficacy 32. Although TGFβ inhibition alone is not a good strategy for cancer treatment, targeting TGFβ signaling may increase the efficacy of chemotherapeutic agents by normalizing the ECM and tumor blood vessels, suppressing EMT, and eliminating CSCs. Therefore, targeting TGFβ signaling is a rational strategy for increasing chemotherapy efficacy. We 33 and others 34-50 have combined multiple TGFβ inhibitors with chemotherapeutic drugs, achieving positive results not only in mouse tumor models but also in solid tumor clinical trials 51-53.

Herein, we summarize the progress in targeting TGFβ signaling to enhance cancer chemotherapy efficacy. We will focus on the distinct roles of TGFβ signaling in the pharmacokinetics and pharmacodynamics (PK/PD) of chemotherapeutic agents. We briefly describe the TGFβ signaling pathway, the roles of TGFβ signaling in the TME and cancer cells, and TGFβ blocking agents in clinical trials. Then, we highlight the most recent progress in targeting TGFβ signaling to enhance chemotherapy efficacy by normalizing the ECM, modulating the tumor vasculature, suppressing EMT, and eliminating CSCs. Finally, we outline the current challenges in targeting TGFβ signaling and discuss future directions for the rational combination of TGFβ blocking agents with chemotherapeutic drugs.

## TGFβ signaling

**Figure 1:**
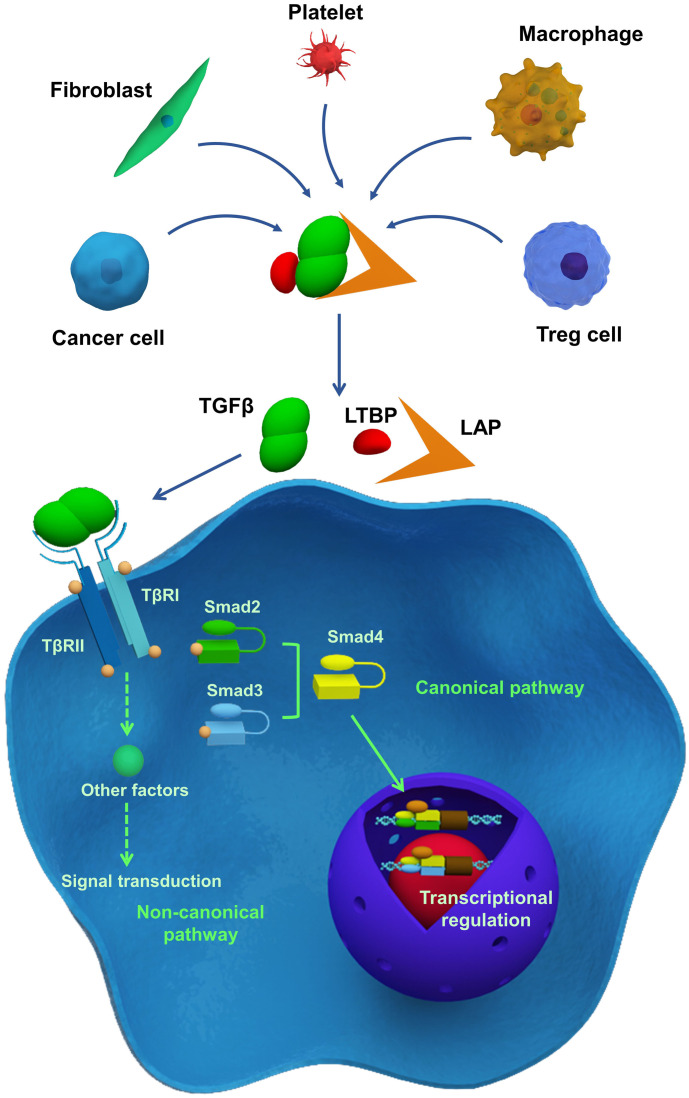
Essentials of TGFβ signaling pathway. TGFβ is secreted by different cells and stored in ECM as a latent form, which interacts with latency-associated peptide (LAP) and latent TGFβ-binding protein (LTBP). Following their activation, TGFβ receptors transmit signals via the SMAD-dependent canonical pathway or SMAD-independent non-canonical pathway, thereby regulating gene and protein expression and cellular function.

TGFβ signaling is essential in development, homeostasis, tissue regeneration, and immune tolerance. However, TGFβ also plays a role in tumorigenesis, tumor progression, and metastasis 11. Depending on the cell types and cellular contexts, TGFβ signaling can have different, and sometimes even opposite, functions 2. Though TGFβ generally inhibits cell proliferation, it can promote cell growth under certain conditions. TGFβ can enhance stem cell pluripotency, but it can also induce stem cell differentiation. TGFβ can induce apoptosis in pre-malignant cells while promoting tumor progression and metastasis 54. Therefore, understanding TGFβ signaling is critical, and current studies have been reviewed in detail elsewhere 23, 55. Here, we briefly summarize the key elements of TGFβ signaling (**Figure 1**).

TGFβ ligands initiate TGFβ signaling. There are three human TGFβ isoforms: TGFβ1-3 54, 56. These ligands are overproduced by cancer cells, Treg cells, fibroblasts, macrophages, and platelets, and are stored in their latent forms in the ECM 24. Latent TGFβ ligands form a homodimer, which interacts with latency-associated peptide (LAP), and latent TGFβ-binding protein (LTBP; **Figure 1**). Latent TGFβ can be activated by matrix metalloproteinases (MMP) 2 or 9, or thrombospondin 1 (THBS1) 57. Additionally, αVβ6 integrin is involved in TGFβ activation and release through binding to the RGD motif in LAP aided by the contractile force generated by myofibroblasts or CAFs 58-60. When TGFβ ligands are activated, they interact with type II TGFβ receptors (TβRII), which subsequently recruit and phosphorylate type I TGFβ receptors (TβRI), thereby propagating downstream signaling. As illustrated in **Figure 1**, the phosphorylation of TβRI activates downstream signaling through either the SMAD-dependent canonical pathway or the SMAD-independent non-canonical pathway 61. In the canonical pathway, receptor-specific SMADs (R-SMAD), including SMAD2 and SMAD3 can be phosphorylated by activated TβRI. Phosphorylation of R-SMAD induces its oligomerization with other mediators (SMAD4), and the formation of a SMAD complex. Then, the complex translocates to the nucleus and interacts with other co-factors, resulting in target gene expression 62. The SMAD-independent, non-canonical pathway involves the activation of other signaling pathways through interactions between the activated TGFβ receptor complex and tumor necrosis factor (TNF) receptor-associated factor (TRAF) 4 or TRAF6, TGFβ-activated kinase 1 (TAK1), p38 mitogen-activated protein kinase (MAPK), Rho GTPases, extracellular signal-regulated kinase (ERK), c-jun N-terminal kinase (JNK), or nuclear factor-κB (NF-κB) 63. The non-canonical- and SMAD-dependent canonical pathways can also regulate each other. Also, both the SMAD-dependent canonical pathway and the SMAD-independent non-canonical pathway can be regulated by other signaling pathways, including the Akt-PI3K, Wnt, Hedgehog (HH), Notch, interferon (IFN), and Ras signaling 55.

**Figure 2:**
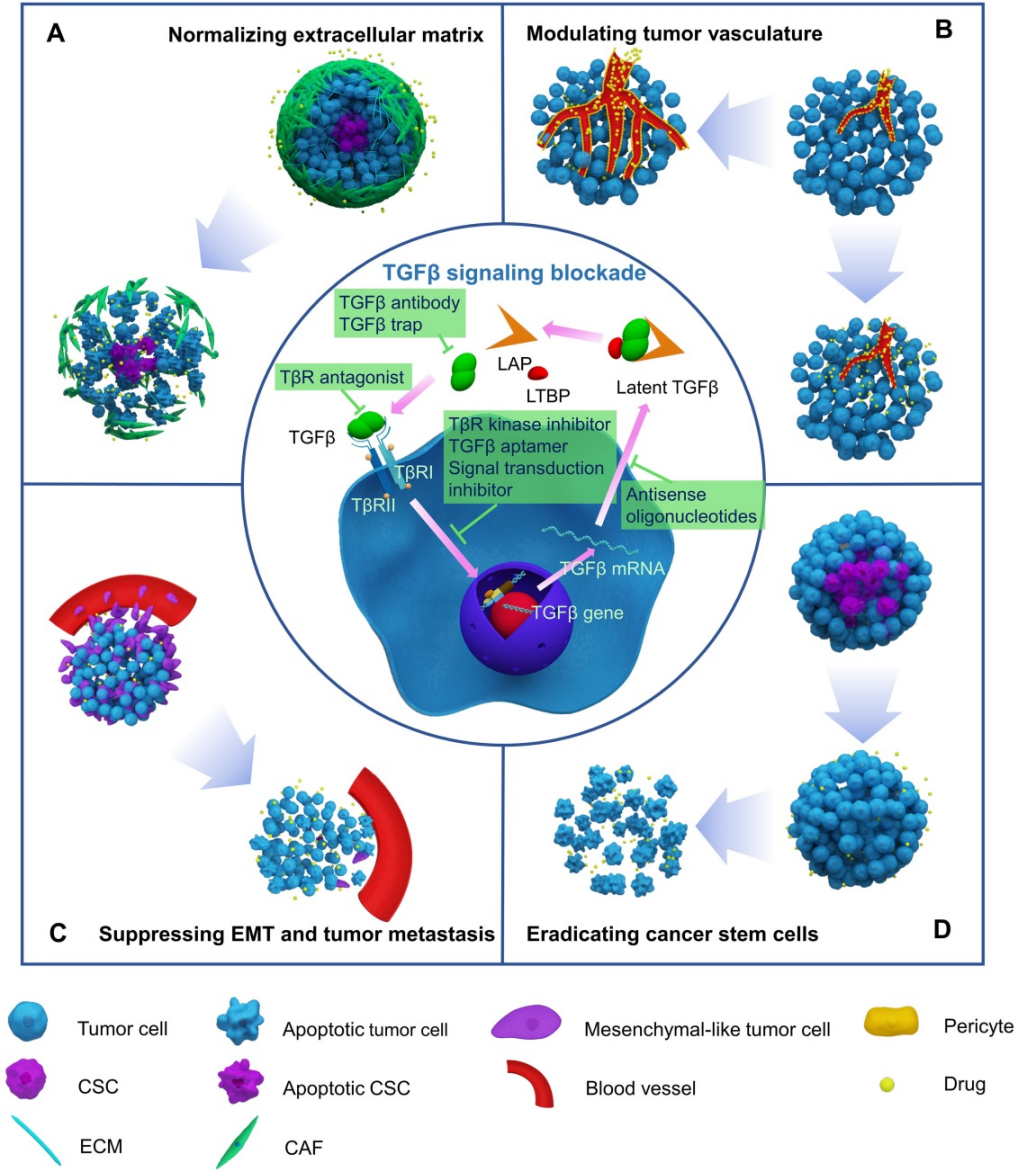
Blocking TGFβ signaling to enhance chemotherapy efficacy by normalizing extracellular matrix (A), modulating tumor vasculature (B), suppressing EMT and tumor metastasis (C), and eradicating cancer stem cells (D).

With increased understanding of the TGFβ pathway, two general strategies have been developed to block TGFβ signaling. The first strategy involves interrupting the interaction between TGFβ ligands and receptors by using TGFβ antibodies, TGFβ trap, or TGFβ receptor antagonists, while the other strategy interferes with the downstream pathway with signal transduction inhibitors (**Figure 2**).

## Roles of TGFβ in the tumor microenvironment

TGFβ can promote the formation of an aberrant TME and is extensively involved in ECM remodeling and tumor angiogenesis. TGFβ increases the expression of ECM-associated genes in tumor stroma cells, induces transformation of fibroblasts to myofibroblasts or CAFs, and enhances ECM accumulation with the help of integrins. TGFβ can affect the tumor vasculature directly or indirectly. TGFβ induces the formation of an immunosuppressive tumor microenvironment and facilitates carcinoma cell escape from immune surveillance. TGFβ downregulates the immune response in the following ways: TGFβ decreases the exposure of antigen-presenting cells (APCs) to antigens and inhibits their function. TGFβ also enhances Treg activation, while inhibiting Th1, Th2, and NK cells 64. Further, TGFβ is essential for T cell homeostasis by maintaining naïve T cells 65, 66. Moreover, TGFβ also impairs the immune response by promoting ECM accumulation and preventing the infiltration of immune cells, including T cells, NK cells, and neutrophils into tumor tissues 67. The role of TGFβ in regulating the tumor immune microenvironment is different from that in chemotherapeutic drug delivery and chemosensitization. Therefore, in this section, we will only discuss the impact of TGFβ signaling on the tumor matrix and blood vessels, and not the role of TGFβ in antitumor immunity and immune therapy. The role of TGFβ signaling in establishing an immunosuppressive tumor microenvironment and in cancer immunotherapy has been reviewed in detail elsewhere 23-25, 68, 69.

TGFβ signaling enhances ECM deposition by transforming the phenotype of fibroblasts 70. TGFβ induces normal fibroblasts to differentiate into myofibroblasts or CAFs, which act as the primary source of extracellular matrix proteins 1, 71. Further, TGFβ induces the expression of ECM-associated genes in epithelial cells, including collagen type 1 α1 (COL1A1), COL4A1, MMP2 and 9, and lysyl oxidase homologue 4 (LOXL4) 72-77. Activation of ECM-associated gene expression leads to increased production of matrix proteins, including collagen, fibronectin, tenascin, and proteoglycans. MMPs degrade collagen, while lysyl oxidase (LOX) crosslinks collagen proteins to remodel the ECM and form a dense collagen network 78-81. In addition to TGFβ-induced MMP overexpression, the levels of plasmin and proteases are also increased. This resulted in the release of TGFβ ligand trapped as a latent form in the ECM and activation of TGFβ signaling. Activated TGFβ signaling then promotes the expression of MMPs, plasmin, and proteases, thereby establishing a positive regulatory loop to modulate the ECM 82. TGFβ also promotes matrix stiffening through activation of integrins. In NIH 3T3 fibroblasts, TGFβ1 increases COL1A2 promoter activity, which is mediated by αvβ3-integrins, to enhance matrix protein production 83. In metastatic mouse breast cancer cells, αvβ3-integrins can upregulate proteinase inhibitors, such as plasminogen activator inhibitor 1 (PAI-1), to decrease ECM degradation 84. Therefore, TGFβ overexpression leads to ECM accumulation and increased mechanical stiffness of tumor tissues 85, 86, thereby creating a physical barrier to chemotherapeutic drug delivery.

In addition to modulating the ECM, TGFβ also acts as a potent mediator of tumor angiogenesis. TGFβ directly regulates tumor blood vessel structure and function through TGFβ/activin-receptor like kinase-1 (ALK1) or TGFβ/ALK5 signaling in endothelial cells and pericytes. ALK1 activation stimulates pericyte recruitment and endothelial cell proliferation and migration, whereas ALK5 activation exerts an opposite effect to maintain blood vessel stabilization 87. Additionally, TGFβ can increase the expression of vascular endothelial growth factor (VEGF) and TNF-α, which can promote tumor angiogenesis by stimulating the proliferation and migration of endothelial cells 88. TGFβ can also induce angiogenesis indirectly by stimulating cytokine release by other cells. For example, TGFβ promotes the secretion of angiogenic cytokines by monocytes, thereby stimulating blood vessel formation 89. TGFβ can further modulate the tumor vasculature by inducing MMP2 and 9, which are conducive to endothelial cell migration and capillary formation. TGFβ can also upregulate the expression of paracrine factors, such as hepatocyte growth factor (HGF), chemokine (C-X-C motif) ligand 1 (CXCL1), and CXCL16, to induce the invasion of blood vessels into adjacent epithelia 80. In turn, these factors stimulate TGFβ expression, thereby resulting in a positive feedback loop to stimulate tumor angiogenesis.

Overall, TGFβ promotes the ECM component deposition and tumor angiogenesis, leading to the formation of a complex TME. Excessive ECM and abnormal tumor vasculature hamper drug penetration and accumulation in tumor tissues by inhibiting not only interstitial transport but also trans-vascular delivery. TGFβ induces ECM component accumulation and the formation of a dense physical barrier that hinders drug delivery. Simultaneously, TGFβ overexpression promotes tumor angiogenesis, leading to aberrant tumor vasculature. These mechanisms lead to decreased blood perfusion and compromised drug delivery to tumor tissues. Thus, TGFβ signaling is a major cause of poor drug delivery. Targeting TGFβ signaling to normalize tumor ECM and modulate the tumor vasculature is therefore expected to increase drug penetration and accumulation in solid tumors.

## Roles of TGFβ in cancer cells

TGFβ signaling exerts two opposite effects in cancer cells. TGFβ signaling can suppress tumorigenesis by inducing apoptosis of pre-malignant cells and inhibiting the proliferation of cancer cells 4, 90. However, in late-stage malignancies, TGFβ facilitates tumor progression and metastasis by inducing EMT, promoting CSCs initiation and proliferation, and maintaining CSCs 91. Next, we will discuss the dual role of TGFβ signaling in cancer cells.

TGFβ can act as a tumor suppressor in pre-malignant cells. TGFβ promotes apoptosis through TGFβ/SMAD signaling and downstream effectors including TGFβ-inducible early-response gene (TIEG1), SH2 domain-containing inositol-5-phosphatase, death-associated protein kinase 1 (DAPK1), and B-cell lymphoma 2 (BCL2) 92. TGFβ signaling inhibits cancer cell proliferation by inducing cell cycle arrest through regulation of cyclin-dependent kinases (CDK), CDK inhibitors, and cyclins 93, 94. In addition to the direct impact on cell cycle proteins, TGFβ-induced downregulation of myc can also result in cell cycle arrest in G1 and S phases through activation of p21 and p15 95-97. These events inhibit cancer cell proliferation and promote apoptosis.

On the other hand, TGFβ signaling can promote invasion and metastasis of late-stage cancer cells 98. During tumor progression, elevated expression of TGFβ ligands can induce EMT, resulting in tumor invasion and metastasis. In hepatocellular carcinoma, TGFβ/SMAD signaling promotes tumor metastasis 99, 100. Protein tyrosine phosphatase receptor ε (PTRPε) interacts with TβRI and induces the recruitment of SMAD3 to TβRI in a tyrosine phosphatase-dependent manner. PTRPε continually activates SMAD3 and promotes EMT, thereby promoting tumor metastasis 101. During EMT, epithelial cells transform into a mesenchymal-like phenotype, by downregulating the expression of several proteins, including E-cadherin, which is necessary for cell-basement membrane and cell-cell adhesion. This transition results in a more motile cell phenotype and promotes tumor cell metastasis. Concomitantly, EMT-associated transcription factors, including Snail1 and 2, zinc finger E-box-binding homeobox 1 (ZEB1), ZEN2, and lymphoid enhancer-binding factor 1 (LEF1), and other proteins, such as N-cadherin and vimentin, are upregulated. Further, TGFβ promotes cytoskeleton rearrangement, thereby facilitating metastasis 102. Furthermore, TGFβ signaling regulates CSCs initiation and proliferation. Mani et al. demonstrated that human mammary epithelial cells with mesenchymal traits expressed stem-cell markers and acquired the capacity to form mammospheres 103. TGFβ signaling also regulates CSCs function, where the self-renewal capacity and stemness of CSCs are modulated through the cyclin D1-Smad2/3-Smad4 signaling pathway 50.

The dual effects of TGFβ signaling on cancer cells can also manifest in regulating cellular dormancy, which is key to drug resistance. TGFβ signaling can promote tumor cell dormancy as well as facilitate tumor cells escape from dormancy 104. These distinct effects depend on the cellular context, including the availability of TGFβ ligands and receptors. Although the exact mechanisms are unclear, recent studies showed that dormancy regulation is dependent on either canonical 105 or non-canonical TGFβ signaling 106, 107. TGFβ1 induced dormancy in squamous cell carcinoma models 108, 109, but it activated dormant T4-2 breast cancer cells in 3D culture 110. TGFβ1 did not promote dormancy in prostate cancer cells, whereas TGFβ2 induced quiescence in C4-2B4 cells. Knockdown of TβRIII in C4-2B4 cells interfered with TGFβ2-induced cellular dormancy. However, different prostate cancer cell lines had distinct responses to TGFβ2. Following TGFβ2 treatment, C4-2B4, C4-2b, and PC3-mm3 cells, but not 22RV1 and BPH-1 cells, entered a dormant state 111. Non-canonical TGFβ signaling controls cellular quiescence by regulating the Akt/PI3K pathway, and the expression of differentiated embryonic chondrocyte expressed gene 2 (DEC2) 106, 107, 112, 113. TGFβ signaling also promotes tumor latency by modulating the tumor microenvironment through regulation of angiogenesis and immunosuppression 104, 114.

Although TGFβ signaling has opposing effects on cancer cells, tumor progression and dissemination are the outcomes of TGFβ signaling in late-stage cancers 98. Overexpressed TGFβ ligands accelerate EMT and enhance cancer cell stemness, which results in chemoresistance, tumor metastasis, and tumor relapse, and remains the key unresolved clinical issues 115.

## Inhibitors that target TGFβ signaling

Recognizing the tumorigenic roles of TGFβ in late-stage malignancies has accelerated the development of drugs that target TGFβ signaling. A series of inhibitors have been developed to block TGFβ signaling (**Figure 2**). TGFβ inhibitors include neutralizing antibodies that interfere with latent TGFβ activation and suppress ligand-receptor interactions, ligand traps that target TGFβ ligands by sTβRII-Fc or sΒglycan-Fc fusion proteins and prevent ligand-receptor binding, receptor antagonists that target TGFβ receptors and interfere with TGFβ ligands-receptor interactions, TGFβ receptor kinase inhibitors, and antisense oligonucleotides (AONs) that silence target gene expression, TGFβ aptamers that disrupt SMAD protein-protein interactions, and downstream signal transduction inhibitors 116, 117. TGFβ inhibitors are currently in 48 phase II and 6 phase III clinical trials. Both positive and negative clinical outcomes have been observed. Current pre-clinical and clinical studies of different TGFβ inhibitors for cancer therapy are summarized in Table S1.

TGFβ signaling blockade can result in improved OS and progression-free survival (PFS). For instance, administration of the TβRI kinase inhibitor galunisertib (LY2157299) was demonstrated to be effective as a monotherapy or in combination with chemotherapy in phase II clinical trials for patients with advanced hepatocellular carcinoma (HCC), unresectable pancreatic cancer, and metastatic breast cancer 100, 101. Forty patients with advanced HCC who had progressed or were ineligible to receive sorafenib were enrolled in a phase II galunisertib trial (NCT01246986) 51. Significantly, in 74% of patients serum TGFβ1 decreased by 20% and the median OS was 21.8 months, whereas the median OS was 7.91 months for patients with less than 20% reduction in serum TGFβ1. In a phase II, double-blind study in patients with unresectable pancreatic cancer that compared treatment of galunisertib and gemcitabine (GG) vs gemcitabine and placebo (GP), galunisertib treatment was more beneficial for patients with lower TGFβ1 levels (NCT01373164) 52. For patients with TGFβ1 levels lower than 4224 pg/mL (n = 117), the median OS was 10.9 months in the GG group, which was 3.7 months longer than that of patients in the GP group. Serious adverse events occurred in 54.37% (56/103) and 50.00% (26/52) of GG and GP group patients. Fresolimumab (GC1008), a pan-TGFβ isoform neutralizing antibody, was tested in a phase II clinical trial for patients with metastatic breast cancer (NCT01401062) 118. Concomitant fresolimumab treatment and radiotherapy were well tolerated. Patients treated with the higher dose (10 mg/kg) had a favorable systemic immune response and significantly higher median OS than patients treated with the lower dose (1 mg/kg). In addition to these specific TGFβ inhibitors, the anti-hypertension drug, losartan, and anti-fibrotic drugs, including tranilast and pirfenidone, have also been reported to suppress TGFβ signaling. Losartan inhibits TGFβ1 activation by reducing thrombospondin-1 (TSP-1) expression 38. A phase II study in patients with locally advanced pancreatic ductal adenocarcinoma showed that FOLFIRINOX (fluorouracil, leucovorin, oxaliplatin, and irinotecan) and losartan therapy followed by individualized chemoradiotherapy resulted in a high R0 resection rate and higher survival rates (NCT01821729) 53. The median PFS was 17.5 months for 49 eligible patients, while the median OS was 31.4 months.

While these clinical trial results indicate that blocking TGFβ can be beneficial for cancer treatment, TGFβ inhibitors encounter numerous translational challenges. Because TGFβ signaling plays a critical role in maintaining homeostasis of normal tissues, safety is a concern. To that end, the safety and efficacy of fresolimumab were tested in a phase II study of 14 patients with malignant pleural mesothelioma (NCT01112293). Despite the small sample size and lack of post-treatment tumor biopsies, this study showed that 85.71% (12/14) and 42.86% (6/14) of patients experienced adverse events and serious side effects after a 3-week fresolimumab treatment cycle. The anti-ALK-1 monoclonal antibody PF-03446962 was tested in phase I studies in patients with HCC and other advanced solid tumors (NCT00557856, NCT01337050) 119-121. The good safety profile and antitumor efficacy supported further studies of PF-03446962 in patients with HCC and advanced solid malignancies. However, in a phase II study, PF-03446962 failed to demonstrate a therapeutic effect in patients with malignant pleural mesothelioma who were treated with a median of four cycles (range 1-12) of PF-03446962 (NCT01486368) 122. Trabedersen (AP12009) is a TGFβ2-specific AON that silences TGFβ2 gene expression to attenuate TGFβ signaling. In a phase II study in patients with pancreatic carcinoma, advanced malignant melanoma, and colorectal carcinoma, treatment with trabedersen improved OS (NCT00844064) 123. Trabedersen monotherapy was safe and well-tolerated, and the maximum tolerated dose (MTD) was established as 160 mg/m^2^/day. The median OS for patients with pancreatic carcinoma treated with escalating trabedersen doses lower than MTD was 13.4 months. Unfortunately, a subsequent phase III clinical trial of trabedersen in patients with anaplastic astrocytoma or secondary glioblastoma was terminated due to insufficient patient recruitment (NCT00761280).

Results from existing clinical trials suggest that blocking TGFβ signaling could provide clinical benefits. However, treatment with TGFβ inhibitors alone resulted in severe adverse effects and low antitumor efficacy. Therefore, further studies are needed to ensure that TGFβ inhibitors are safe and effective for clinical use.

## Targeting the TGFβ pathway to enhance chemotherapy

TGFβ signaling-induced deposition of ECM components, tumor angiogenesis, EMT, and cancer cell stemness can inhibit chemotherapy efficacy. Thus, blocking TGFβ signaling is a promising solution to enhance the efficacy of chemotherapy. Inhibition of TGFβ signaling can reverse EMT and prevent tumor metastasis, impair CSCs stemness, normalize the ECM, and modulate tumor blood vessels. Combining TGFβ inhibition and chemotherapy can result in synergistic effects, as shown in numerous cancer models, including breast, liver, pancreatic, and colon cancers (**Table 1** and Table S1). Each work has delineated the role of targeting TGFβ signaling for enhanced chemotherapy (**Figure 2**).

### Normalizing the extracellular matrix

Suppressing TGFβ signaling can lead to ECM remodeling, improved drug penetration into the tumor parenchyma, and enhanced chemotherapy efficacy (**Figure 2A**). To efficiently eradicate cancer cells, chemotherapeutic drugs have to accumulate in tumor tissues and penetrate deep into the tumor parenchyma to achieve a homogenous distribution. Although nanoparticles with a diameter around 100 nm can effectively deliver chemotherapeutic drugs to tumor tissues based on the enhanced permeability and retention (EPR) effect 124, 125, most of the encapsulated cargo remains in superficial tumor regions, and cannot eliminate cancer cells in the tumor core. Excessive ECM forms a dense barrier and impedes nanoparticle penetration into the tumor interior. Even though small-molecule drugs are released from nanoparticles, they are impeded by the physical barrier created by the dense ECM 126-129. CAFs are the primary source of extracellular matrix in solid tumors, while their functions are regulated by TGFβ signaling. Initially, TGFβ induces fibroblasts to transform into myofibroblasts or CAFs, which synthesize and secrete ECM proteins, such as collagen, leading to dense ECM deposition upon Smad3 pathway activation 130. In addition, CAFs secrete MMPs to degrade collagen and remodel the ECM 77. Furthermore, in response to high levels of TGFβ ligands in tumor tissue, CAFs, and other stromal cells overexpress collagen cross-linking proteins, such as LOX 79, 81. Together, CAFs create a dense physical barrier that hampers drug penetration. Therefore, targeting CAFs by blocking TGFβ signaling may restore the ECM and increase chemotherapy efficacy 23, 31, 131.

The anti-fibrotic drug tranilast can normalize the ECM and enhance cancer chemotherapy efficacy. Tranilast at 30-300 μM inhibited the collagen synthesis by fibroblasts derived from keloids and hypertrophic scars. The inhibitory effects of tranilast on collagen synthesis were attributed to decreased TGFβ1 released by fibroblasts 132, and the inhibition of TGFβ1 effects on fibroblasts 133. Because of these effects, tranilast has been used to inhibit TGFβ and restore a normal ECM to improve chemotherapy efficacy. Papageorgis et al. showed that tranilast and chemotherapeutic drug administration in mouse 4T1 and human MCF10CA1a xenografts resulted in ECM remodeling due to reduced collagen and hyaluronan levels 34. Following tranilast treatment, collagen contents were reduced by 20% and 25% in 4T1 and MCF10CA1a, and hyaluronan decreased by 40% and 63% in the two different models, respectively, leading to reduced solid stress and interstitial fluid pressure (IFP). Consequently, tumor blood vessels were decompressed, increasing vessel diameter by 10-15% and blood perfusion by 50-60%. Moreover, this study illustrated that tranilast promoted drug delivery in a size-independent manner by using doxorubicin (DOX, with a diameter less than 1 nm), Abraxane^®^ (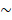
10 nm), and Doxil^®^ (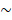
100 nm). Mechanistically, tranilast facilitated ECM remodeling by reducing TGFβ1 expression, thereby inhibiting Smad2/3 phosphorylation, and suppressing TGFβ-mediated expression of COL1A1, connective tissue growth factor (CTGF), and hyaluronan synthase 2 (HAS2) in MCF10CA1a tumor xenografts. Therefore, tranilast markedly improved the antitumor efficiency of Doxil^®^ and the survival rates of both 4T1- and MCF10CA1a- tumor xenograft-bearing mice. The efficacy of this combination therapy was enhanced by adding immune checkpoint inhibitors (PD-1/CTLA-4 blocking antibodies) because the tranilast-mediated tumor ECM remodeling facilitated T cell infiltration into the tumor parenchyma 35.

Pang et al. designed a two-stage therapy with tranilast and docetaxel micelles (DTX-Ms) 36. This two-stage therapy reduced α-SMA-positive CAFs (active CAFs) by 64.8%, decreased IFP by 51.7%, normalized microvessels, improved blood perfusion level by 2.42 times relative to DTX-Ms treatment alone, and promoted drug penetration and retention in tumors. However, this two-stage therapy resulted in a modest increase in antitumor efficacy as compared with concomitant administration of tranilast and DTX-Ms. These studies showed that tranilast could normalize the aberrant ECM deposited by CAFs, alleviate intra-tumoral solid stress and fluid pressure, and decompress tumor blood vessels. In turn, this improved small-molecular chemotherapeutic drugs and nanomedicine delivery and increased their antitumor efficacy.

Pirfenidone (PFD), a novel anti-fibrotic agent, can also restore the ECM and enhance chemotherapy efficacy. PFD exerts its anti-fibrotic effects by downregulating TGFβ expression, inhibiting pSmad2/3, and consequently, suppressing fibroblast proliferation 134, 135. In a triple-negative breast cancer (TNBC) model, PFD treatment in combination with DOX depleted CAFs and inhibited tumor growth 136. Polydorou et al. showed that PFD decreased hyaluronan levels by downregulating TGFβ1, COL1A1, COL3A1, HAS2, and HAS3 expression in MCF10CA1a xenografts 41. Administration of 500 mg/kg PFD in 4T1 tumor-bearing mice resulted in a significant 50% reduction in hyaluronan and a 60% increase in blood perfusion as compared with controls. Because of its effect on hyaluronan levels, PFD decreased tumor opening and tumor stiffness, and reduced IFP in both MCF10CA1a and 4T1 xenografts, suggesting that PFD could reduce the physical barriers in solid tumors. Accordingly, PFD increased DOX delivery to tumors by approximately 50%, leading to significant inhibition of tumor growth in both models. Indeed, both tranilast and PFD treatment resulted in significant ECM remodeling. However, their mechanisms of action are not fully understood. They confirmed that tranilast treatment reduced tumor-associated, α-SMA-positive CAFs. However, Smad2/3 phosphorylation levels and the expression of ECM-associated genes were measured in tumor tissues rather than CAFs. The direct effects of tranilast and PFD on CAFs are largely elusive. While tranilast and PFD have been widely prescribed as anti-fibrotic drugs, the benefits of tranilast or PFD in combination with chemotherapeutic drugs remain to be demonstrated in clinical trials. It is unclear whether these drugs could affect the tumor-associated ECM in patients with different solid malignancies.

Losartan can also suppress TGFβ signaling, reduce ECM component production, and promote drug delivery. In SKOV3ip1 and Hey-A8 xenograft models, losartan decreased αSMA-positive CAFs 39 by downregulating the TGFβ1 activator TSP-1. As a result, the expression levels of profibrotic genes, including *Col1*,* Has1-3*,* Tgfb1*, and *Ctgf*, were reduced in CAFs 37. Losartan destabilized the ECM by reducing CTGF levels 38. Therefore, losartan can act as a dual inhibitor of stromal collagen and hyaluronan to enhance drug penetration. These studies were pioneered by Jain et al. 37, 38, 53, who found that pretreatment with losartan reduced the ratio of αSMA-positive CAFs, and collagen and hyaluronan levels in E0771- and AK4.4 cell-derived tumors 37. The tumors had small tumor openings indicating a reduction in solid stress. Furthermore, the alleviation of solid stress by losartan decompressed tumor blood vessels. Accordingly, the perfused vessel fraction increased from 23% to 43% in E0771 and from 21% to 45% in AK4.4 tumors, leading to enhanced drug penetration and oxygen delivery to both tumor types. Losartan also improved drug delivery efficiency and chemotherapy efficacy in other tumor models. For instance, in ovarian cancer models, losartan treatment facilitated drug diffusion into tumor tissues and enhanced the efficacy of paclitaxel in SKOV3ip1 and Hey-A8 xenograft models 39. Notably, mathematical models have been developed to provide a rationale for the enhanced drug delivery. According to the model, decreased tumor solid stress could enhance drug distribution within the entire tumor. However, drug distribution and penetration depth remain to be determined experimentally. In Mu89 and HSTS26T pancreatic tumor models, the combination of losartan and Doxil^®^ reduced tumor sizes by 50% 38. In a 4T1 breast cancer model, Zhang et al. demonstrated that the infiltration of Evans Blue dye in tumors increased by 21.0% after administration of losartan at 40 mg/kg for 14 days. Treatment with losartan and PTX loaded pH-sensitive cleavable liposome (PTX-Cl-Lip) reduced tumor growth by 59.8%, as compared with PTX-Cl-Lip treatment alone (37.8%) in tumor-bearing mice 40. Like tranilast and PFD, losartan normalized the aberrant ECM, alleviated intra-tumoral solid stresses, decompressed tumor blood vessels, and promoted drug delivery. However, unlike tranilast and PFD, losartan downregulated the CAF activity indirectly by inhibiting TGFβ ligand activation. In these studies, the regulation of ECM related genes by losartan was evaluated in murine CAFs isolated from orthotopic AK4.4 pancreatic tumors. In addition to enhancing chemotherapy efficacy in animal models, losartan treatment also improved OS and PFS in clinical settings. Clinical benefits of FOLFIRINOX and losartan therapy were recently reported in a phase II study (NCT01821729) 53.

In conclusion, tranilast, PFD, and losartan can inhibit TGFβ signaling and promote drug accumulation and penetration in tumor tissues. The attenuated TGFβ signaling results in a decrease in collagen and hyaluronan levels, leading to ECM remodeling, and improved drug delivery (Table 1). The reduced density of the tumor-associated ECM alleviates intra-tumoral solid stress, and leads to microvessel decompression and enhanced blood perfusion. These changes improve drug interstitial and transvascular transport and promote uniform drug distribution and penetration into the tumor parenchyma, thereby enhancing antitumor efficacy. In addition to improving drug delivery efficiency, targeting TGFβ signaling to normalize the ECM can also have indirect effects.

Heterogeneous drug distribution within the tumor can reduce chemotherapy efficacy, and accelerate EMT. Therefore, normalizing the ECM can prevent the adverse effects caused by EMT, including increased cancer cell stemness, chemoresistance, and metastasis. In addition, the remodeled ECM allows for tumor blood vessel decompression, increased blood perfusion, and reduced tumor hypoxia.

### Modulating tumor vasculature

Targeting TGFβ signaling can also modulate the tumor vasculature, thereby enhancing drug delivery and antitumor efficacy (**Figure 2B**). In addition to mediating blood vessel decompression through ECM remodeling, blocking TGFβ signaling can directly regulate the tumor vasculature by affecting perivascular cells. However, depending on the cellular context, two opposite outcomes on vascular integrity have been reported by using different TGFβ inhibitors.

LY364947, a TGFβ receptor I inhibitor, decreased endothelial pericyte coverage in tumor neovasculature, leading to increased drug extravasation into the tumor parenchyma, and improved drug accumulation via the EPR effect 31, 137. Kano et al. demonstrated that systemic administration of a low dose of LY364947 (1mg/kg) in a BxPC3 xenograft model damaged tumor vascular integrity and strengthened the EPR effect of long-lasting nanomedicine, thereby enhancing its accumulation in tumor tissues 42. Further, LY364947 in combination with DOX-loaded polymeric micelles (micelle DOX) significantly inhibited tumor growth, whereas micelle DOX monotherapy had a negligible antitumor effect. Cabral et al. also showed that vascular integrity disruption induced by TGFβ blockade could enhance the accumulation of nanomedicines with diameters around 100 nm in tumor tissues 43. They confirmed that micelles with diameters of 30, 50, 70, and 100 nm penetrated hyperpermeable murine colon adenocarcinoma 26 (C26)-cell derived tumors, whereas only 30 nm micelles could penetrate the poorly permeable pancreatic adenocarcinoma BxPC3-cell derived tumors. However, LY364947 administration increased the tumor blood vessel permeability in poorly permeable tumors. Consequently, following LY364947 treatment, 70 nm micelles displayed comparable distribution to 30 nm micelles in BxPC3-cell derived tumors. Both types of micelles achieved ~20% of V_max_ at 40 μm from the blood vessels at 1 h post-administration and over 40% of V_max_ at 100 μm from the blood vessels at 24 h. The notion that removing endothelial pericytes could improve nanotherapeutic accumulation, and enhance drug penetration into the tumor parenchyma was also supported by Meng et al. who used two-wave nanotherapy to improve tumor targeting of gemcitabine-loaded nanoliposomes. TGFβ signaling was inhibited 2 h post-administration of LY364947-loaded polyethyleneimine (PEI)/polyethylene glycol (PEG)-coated mesoporous silica nanoparticles (MSNPs). Both pericyte differentiation and their attachment to endothelial cells were attenuated. By using Si elemental analysis, the authors demonstrated that about 7% of LY364947-MSNPs were retained at the tumor site 60 h post-administration, whereas less than 0.7% of MSNPs without LY364947 remained in tumor tissues. Importantly, this allowed the nanomedicine to accumulate in tumor tissues effectively. Fluorescence intensity measurements revealed that pretreatment with LY364947-MSNP led to tumor accumulation of ~7% of near-infrared tag-labeled nanoliposomes, which was 4-fold higher than that of nanoliposomes alone. Furthermore, nanoliposome intratumoral distribution was improved, leading to tumor growth inhibition of BxPC3 xenografts 44. These studies showed that administration of low dose LY364947 could reduce pericyte coverage and augment the tumor blood vessel openings. The incomplete vascular structure enhanced the EPR effect and improved drug accumulation in tumor tissues, while also facilitating intratumoral drug distribution due to drug leakage from blood vessels. Together, these mechanisms improved the efficacy of chemotherapeutic drugs.

In striking contrast, TGFβ signaling can also negatively regulate pericyte recruitment during blood vessel stabilization 138. Therefore, TGFβ signaling inhibition increased pericyte coverage of tumor blood vessels. In 4T1- and MDA-MB-231cell-derived tumors, Liu et al. used both genetic (overexpression of a soluble TGFβ type II, sTβRII) and pharmacologic (a TGFβ neutralizing antibody, 1D11) approaches to block TGFβ signaling. They found that the colocalization of NG2 (a pericyte marker) and CD31 (an endothelial cell marker) increased 1.3-1.7 times post-treatment 45. Critically, blood perfusion and intratumoral drug distribution improved due to the improved function of the tumor vasculature. This work highlights that suppressing TGFβ signaling can improve the structure and function of tumor blood vessels. In addition to the direct regulation of blood vessels, 1D11 and sTβRII treatment significantly decreased collagen I content in both tumor types. These observations are consistent with the effects of other TGFβ inhibitors, including losartan, tranilast and PFD, on tumor ECM. Therefore, improvements in vascular integrity and ECM-remodeling mediated blood vessel decompression contributed to increased blood perfusion. In turn, this led to inhibition of tumor growth and metastasis following treatment with a conventional chemotherapeutic drug (DOX) and a nanotherapeutics (Doxil^®^).

These studies showed that targeting TGFβ signaling modulated the structure and function of the tumor vasculature, thereby increasing the drug delivery efficiency and antitumor efficacy of both conventional chemotherapeutic drugs and nanotherapeutics. However, these studies highlighted that the effects of TGFβ signaling inhibition on pericytes and tumor angiogenesis are tumor type-dependent and that the underlying mechanisms have not been elucidated. TGFβ signaling regulates tumor angiogenesis by activating TβRI receptors, including ALK1 and ALK5. Activation of the endothelial cell-restricted ALK1 stimulates endothelial cell proliferation and migration, and pericyte recruitment. However, the activation of ALK5 inhibits pericytes proliferation and migration, and stimulates pericyte differentiation, leading to blood vessel stabilization 85, 139. Therefore, the effect of TGFβ signaling inhibition on endothelial pericyte coverage is dependent on the cellular context. Different TGFβ inhibitors and approaches may yield contradictory results. LY364947 is a potent ATP-competitive inhibitor of ALK5, with an IC_50_ of 59 nM and 7-fold greater selectivity over TβRII. It can decrease endothelial pericyte coverage in BxPC3 xenografts 42, 44. In contrast, 1D11 treatment or overexpression of sTβRII increased pericytes coverage in 4T1- and MDA-MB-231 cell-derived tumors 45. LY364947 preferentially inhibits ALK5 rather than TβRII at a low dose. However, 1D11 and sTβRII interfere with TGFβ ligands and TβRII interactions and affect the downstream signaling, thereby inhibiting ALK1 in endothelial cells and ALK5 in pericyte at the same time. TGFβ inhibitor dosing might also affect the results. The LY364947 systemic administration dose is 1 mg/kg 42-44, which is lower than the intraperitoneal administration doses of 5 or 25 mg/kg 140, 141. Because of the low dose, the effects on tumor cells and ECM components were not significant. Further, BxPC3 cells lack functional Smad4, and their response to TGFβ ligands is attenuated as compared with other cancer cell types 42. Therefore, LY364947 exerts its effects directly on tumor blood vessels and not by regulating tumor cells or the ECM. In contrast, administration of 1D11 at 5 mg/kg inhibited cancer cell proliferation and induced apoptosis, suggesting that it may have direct effects on tumor cells, tumor-associated ECM, and angiogenesis 45.

Different tumor microenvironment characteristics might also be responsible for the conflicting outcomes. Human pancreatic adenocarcinoma BxPC3 cell-derived tumors are poorly vascularized 137, 142, unlike those derived from murine 4T1 and human MDA-MB-231 breast cancer cells. These differences in vascularity may significantly impact on the effects of TGFβ signaling inhibition. However, the effect of TGFβ concentration on pericyte regulation appears to be negligible. Treatment with 1D11 and overexpression of sTβRII markedly increased pericyte coverage in both 4T1 (high TGFβ, 316.9 ± 65.0 pg/mg) and MDA-MB-231 (low TGFβ, 76.3 ± 26.8 pg/mg) cell-derived tumors 45.

Therefore, pericyte regulation via TGFβ signaling inhibition can differ depending on the tumor model. Further studies are warranted to address the conflicting outcomes of these studies conducted by different groups using various tumor models.

### Suppressing chemotherapy-induced EMT and tumor metastasis

EMT plays a central role in tumor progression. During this process, cancer cells gradually lose apical-basal polarity, epithelial cell junctions are disrupted, and the cells transform into a mesenchymal phenotype, thereby acquiring stemness and drug resistance properties that can promote tumor invasion and metastasis 103, 143. In murine breast or skin cancer models, and patient‑derived xenografts, EMT was observed in primary tumors 144, 145 and disseminated tumor cells, prior to the formation of detectable metastases 146. In addition to promoting cancer cell dissemination to distant tissues, EMT also enables disseminated cells to enter a CSC state, which is key for metastasis initiation 147. As discussed above, TGFβ signaling is essential for EMT 48, 148-151. Notably, insufficient chemotherapy can induce EMT by activating TGFβ signaling in various malignancies 152. The efficacy of conventional chemotherapy can be limited by heterogeneous drug distribution in tumor tissues due to excessive tumor ECM deposition, tumor solid stress, and a high IFP 153. Indeed, most drugs accumulate at the tumor parenchyma margin and kill cancer cells that are adjacent to blood vessels. Insufficient chemotherapy doses do not eradicate cancer cells that are far from blood vessels, but instead accelerate their EMT program 154. Fan et al. reported that 4T1 breast cancer cells acquired mesenchymal phenotypes after treatment with DOX at IC_50_ concentration 152, likely due to upregulation of TGFβ1. Similar to DOX, cisplatin, paclitaxel, camptothecin, and 5-fluorouracil also induced TGFβ1 expression in different cancer cells, including 4T1, MDA-MB-231, HeLa, C-4, TOV-21G, OVCAR-3, HT-29, and HCT116p53KO 9, 32, 155. Hence, blocking TGFβ signaling could be a promising strategy for addressing insufficient chemotherapy-induced EMT and tumor metastasis (**Figure 2C**).

TGFβ signaling blockade can suppress insufficient chemotherapy induced EMT program and tumor metastasis. Ren et al. demonstrated that LY2109761, a TβRI/II kinase inhibitor, inhibited metastasis and enhanced chemosensitivity in MG-63 osteosarcoma (OS) cells 46. The mechanism of action of LY2109761 included inhibition of TGFβ signaling and regulation of S100A4, which is a known EMT marker. S100A4 is a calcium-binding protein that is overexpressed in multiple cancer cells and could facilitate their invasion and metastasis by interacting with Smad3 156-158. High levels of S100A4 promoted tumor cell migration and decreased the efficacy of cisplatin. However, combination treatment with LY2109761 restored cisplatin chemosensitivity and inhibited MG-63 cell invasion 46. While LY2109761 treatment reduced S100A4 EMT marker levels, the phenotypic transformation of cancer cells and the relationship between TGFβ signaling, EMT, and tumor metastasis remain to be examined in depth. Furthermore, mechanistic studies have only been performed in cultured cells, and more complex *in vivo* studies are lacking.

Our group illustrated the benefits of combining TGFβ inhibitors with chemotherapy in two animal models. We developed a co-delivery strategy for DOX and LY2157299 (LY) with hydroxyethyl starch-polylactide nanoparticles (DOX/LY@HES-PLA) to suppress insufficient chemotherapy-induced tumor metastasis 33. *In vitro* studies revealed that DOX treatment remarkably increased the active TGFβ1 concentration in 4T1 cell culture medium (DOX group: 112 pg/mL; DOX@HES-PLA group: 104 pg/mL), whereas the combination of DOX and LY reduced TGFβ1 levels to 30 pg/mL, which was comparable to that in control cells (35 pg/mL). With decreased TGFβ1 levels, expression of pSmad2, N-cadherin, and vimentin was reduced, while E-cadherin levels increased, indicating effective inhibition of the EMT process. Consistently, inhibition of chemotherapy-induced EMT was also observed in 4T1 tumor-bearing mice. The average serum TGFβ1 levels were 2742 pg/mL and 2205 pg/mL in DOX and DOX@HES-PLA groups, while TGFβ1 levels in control mice were 1377 pg/mL. Strikingly, DOX/LY@HES-PLA significantly decreased TGFβ1 concentration to 986 pg/mL. However, TGFβ1 concentration in the DOX + LY group was 2269 pg/mL, highlighting the advantage of encapsulating both drugs within the nanoparticles. Because DOX and LY have distinctive physicochemical properties, although DOX and LY had potent EMT-suppressive effect *in vitro*, the drugs did not accumulate in tumor tissues with spatiotemporal synchronization and precision. Metastasis of 4T1 cells was also evaluated in a zebrafish model. Invasive cancer cells were detected in 50% of zebrafish in the absence of TGFβ inhibition, while the percentage was reduced to 20% upon treatment with LY. The number of metastatic cells per zebrafish increased from 15 in the control group to approximately 70 in the DOX-treated groups. Significantly, there were fewer than 10 metastatic cells in zebrafish with LY. In mice bearing 4T1 cell-derived subcutaneous tumors, DOX/LY@HES-PLA had the highest tumor growth inhibition rate (80.7%) as compared with all other groups. Moreover, almost no pulmonary metastatic nodules were observed in the DOX/LY@HES-PLA group, whereas the average number of nodules per lung in other groups was approximately 10. This study highlights that heterogeneous drug distribution inside tumor tissues can lead to insufficient chemotherapy, thereby accelerating EMT, and promoting tumor metastasis by downregulating E-cadherin and upregulating N-cadherin. Moreover, apart from combining TGFβ inhibitors and chemotherapeutic drugs within nanoparticles to reverse EMT, it might be more advantageous to achieve homogenous drug distribution within tumors, as with therapies that promote tumor ECM remodeling. Thereby, cancer cells are eradicated with sufficient chemotherapeutic drug concentrations throughout the tumor. While LY2157299 has been evaluated in several clinical trials (Table S1), the clinical benefits of co-administration LY2157299 and chemotherapy within nanoparticles remain to be demonstrated.

TGFβ signaling inhibition-mediated depletion of collagen I and LOX is another approach for suppressing pulmonary metastasis. Zhang et al. co-administrated losartan and PTX-Cl-Lip to 4T1 tumor-bearing mice, resulting in significant inhibition of primary tumor growth metastases 47. PTX-Cl-Lip-mediated inhibition of primary tumor growth was increased from 45.5% to 63.3% after losartan treatment. Pulmonary metastatic nodules were reduced by 76.4% and anti-metastatic efficacy was enhanced up to 88.2%. Losartan did not affect total TGFβ1 concentration in tumors, but it reduced active TGFβ1 by 18.4%. It was postulated that losartan inhibited tumor metastasis by reducing LOX- and collagen I-mediated integrin signal transduction. However, the relationship between tumor metastasis, collagen I, LOX, and integrin activation in the context of EMT has not been fully elucidated at the cellular and molecular levels. The mechanism losartan-mediated metastasis inhibition is also not known. It is possible that insufficient chemotherapy-induced EMT and metastasis are alleviated by losartan. However, these speculations remain to be confirmed by further studies.

### Eradicating cancer stem-like cells

Targeting CSCs for destruction or irreversible quiescence is critical for the treatment of chemo-resistant cancers 55. Chemo-resistant CSCs with self-renewal and tumor initiation capacities are the main cause of tumor relapse and metastasis after chemotherapy 159. The chemoresistance of CSCs can be due to expression of antiapoptotic proteins and drug transporters, efficient DNA repair, and quiescence 160. Thus, it is important to target CSCs maintenance pathways to sensitize CSCs to chemotherapy. EMT enables cells to convert to a CSC phenotype 147, 161, 162. EMT induced by TGFβ treatment conferred CSCs properties to epithelial cancer cells, including expression CSC-specific cell-surface markers (CD44^high^/CD24^low^) and enhanced sphere formation ability 161. Targeting TGFβ signaling to enhance chemotherapy efficacy in eradicating CSCs is therefore, of great clinical significance (**Figure 2D**).

Regulating the population, self-renewal capacity, oncogenic activities, and chemosensitivity of CSCs in tumors through TGFβ signaling are potential approaches to eradicate CSCs and enhance chemotherapy efficacy. TGFβ signaling is involved in CSCs expansion 147, 161, and its inhibition could reduce the CSC population and enhance the cancer cell susceptibility to chemotherapeutic drugs. Bhola et al. showed that paclitaxel-induced CSCs expansion was regulated by Smad4-dependent expression of IL-8 in triple-negative breast cancers 48. These CSCs could be eliminated by treatment with LY2157299, a TβRII-neutralizing antibody, TR1, and siRNA-mediated Smad4 downregulation. In SUM159 and BT549 cell lines and mouse xenografts, paclitaxel activated TGFβ signaling and increased the CSC population. In two clinical studies, TGFβ signaling- and CSC-associated gene expression increased after chemotherapy in breast cancer patients. Importantly, cancer cells isolated from LY2157299-treated mice had reduced tumor-forming capabilities when they were re-injected into mice after extreme limiting dilution. Though the molecular mechanisms of CSC selection warrant further investigation, this study elegantly establishes a correlation between chemotherapy, autocrine TGFβ signaling, IL-8 expression, and CSC expansion. Thus, this study emphasizes the significance of TGFβ signaling for CSC expansion and highlights the risk of chemotherapy-induced CSC population expansion. CSCs with self-renewing and tumor-initiating properties could lead to tumor relapse and metastasis. Therefore, targeting tumor-initiating cells with TGFβ inhibition after chemotherapy could improve breast cancer treatment outcomes. While LY2157299 has been evaluated in a phase II clinical trial in metastatic breast cancer patients (NCT02538471), the benefits of suppressing CSC by inhibiting TGFβ remain to be determined in a clinical setting.

Importantly, TGFβ signaling is necessary to maintain the self-renewal capacity of CSCs 163. Thus, inducing CSC differentiation by inhibiting TGFβ could prevent chemoresistance. Cyclin D1-Smad2/3-Smad4 is a critical pathway for HCC CSC self-renewal. Cyclin D1 interacts with and enhances TGFβ/Smad signaling to induce the expression of chemoresistance-associated gene ABCB1. Xia et al. used SB431542 (SB), an inhibitor of ALK receptors, to impair CSC self-renewal, CSC marker and stemness gene expression, and chemoresistance, in HCC cell lines (97H and Huh7) and primary cancer cells derived from HCC patients 50. When TGFβ/Smad signaling was abrogated by SB in cyclin D1-expressing spheres, HCC CSC populations and the expression of stemness genes (*NANOG*, *OCT4*, and *SOX2*) were significantly reduced. More than 80% of spheres lost their stemness characteristics, implying that TGFβ inhibition induced cyclin D1-expressing CSC differentiation. In addition, *E-CADHERIN* and *CK19* gene expression increased, whereas *SNAIL 1/2* and *N-CADHERIN* gene expression decreased after treatment with SB. These results highlight that SB treatment can reverse EMT, which is necessary for CSC stemness maintenance. Interestingly, tumor growth was significantly inhibited, and 57% of the tumors were fully eliminated, only when cisplatin was administered after a low dose of SB, but not together with SB. However, the underlying mechanisms remain elusive and warrant further investigation. In addition to cisplatin, SB treatment also significantly sensitized CSCs to other chemotherapeutic agents, including oxaliplatin and doxorubicin. While the association between cyclin D1, Smad 2/3, and Smad 4 has been established and can be an indicator of poor prognosis in HCC patients, the clinical efficacy of SB treatment to induce CSC differentiation, thereby potentiating chemotherapy efficacy remains to be demonstrated.

Aberrant TGFβ tumor suppressor activity can promote CSC stemness and oncogenic potential 164-166. Therefore, restoring TGFβ normal signaling could improve chemosensitivity. Chen et al. demonstrated that the TGFβ tumor suppressor pathway was not functional in toll-like receptor 4 (TLR4)/NANOG-dependent HCC CSCs (CD133^+^ and CD49f^+^) and identified *Yap1* and *Igf2bp3* as novel TLR4/NANOG-dependent genes 49. YAP1 and IGF2BP3 exerted their oncogenic activities by inhibiting Smad3 phosphorylation as well as preventing pSmad3 nuclear translocation. Silencing YAP1 and IGF2BP3 restored TGFβ signaling, reduced stemness gene expression, and sensitized HCC CSCs to rapamycin and/or sorafenib. This study elegantly establishes a novel and interesting relationship between TRL4 and TGFβ, emphasizing the significance of the TGFβ tumor suppressor pathway in preventing the formation of HCC CSCs. However, such reciprocal regulation has only been confirmed in HCC, and it remains to be investigated in breast, lung, and pancreatic cancers, which have distinct pathophysiological characteristics.

TGFβ signaling regulates the expansion, self-renewal capacity, oncogenicity, and chemosensitivity of CSCs 160. Targeting the TGFβ signaling pathway to regulate CSCs in tumors can increase chemotherapy efficacy and prevent tumor recurrence 167. Therefore, the combination of TGFβ signaling modulation and chemotherapy can be a potential strategy for elimination of chemo-resistant CSCs. Since TGFβ has a dual role as a tumor suppressor and tumor promoter, CSCs can be eradicated by either blocking or restoring TGFβ signaling. However, the strategy for TGFβ signaling regulation will depend on the cancer type and will require further studies of the TGFβ signaling pathway at the cellular and molecular levels. CSCs are normally located in the tumor core. As discussed above, inhibition of TGFβ signaling restores the ECM 34, 36-38, 41 and modulates tumor blood vessels 42-45 to promote drug penetration into the tumor parenchyma. This could improve drug delivery (TGFβ inhibitors or chemotherapeutic agents) to CSC-rich areas, leading to enhanced antitumor efficacy.

## Conclusion and Perspectives

In this review, we discuss four benefits of targeting TGFβ signaling to enhance chemotherapy efficacy: (1) normalizing the ECM to enhance drug penetration; (2) modulating the tumor vasculature to promote drug delivery; (3) suppressing insufficient chemotherapy-induced EMT and tumor metastasis; and (4) eradicating CSCs. Though some progress in combining TGFβ inhibitors with chemotherapy for treatment of different cancers in animal studies and clinical trials, multiple scientific challenges remain to be addressed.

Restoring the tumor suppressive effects of TGFβ signaling awaits further investigation. TGFβ has a dual role as a tumor suppressor and tumor promoter. However, genetic mutations in TGFβ promote its oncogenic activity rather than its tumor suppressive effects 5, 168. While most current therapeutic agents (Table S1) inhibit TGFβ signaling, several lines of evidence suggest that restoring TGFβ tumor suppressor activity can be beneficial for cancer chemotherapy. Chen et al. confirmed that restoring TGFβ signaling by silencing YAP1 and IGF2BP3 could reduce stemness gene expression and abrogate chemoresistance in HCC CSCs 49. Copland et al. found that loss of TβRIII and TβRII expression in renal cell carcinoma (RCC) and metastatic RCC, and restoring TβRII and TβRIII expression in UMRC3 cells could restore TGFβ-mediated transcriptional responses, thereby attenuating cancer cell proliferation 169. Further, loss of Smad4 was reported in colorectal 170 and pancreatic cancer 171. LY2109761 blocked the oncogenic effects of TGFβ in Smad4-null MC38 cells, while exogenous Smad4 expression in MC38 cells reverted TGFβ from a tumor promoter to a tumor suppressor 170. The introduction of functional Smad4 into a Smad4-deficient pancreatic cell line (BxPC-3) restored TGFβ-mediated responses 171. These studies highlight a distinct way to restore TGFβ signaling and its tumor suppressive effects. However, different tumor types have distinct pathophysiological characteristics. A thorough context-dependent understanding of TGFβ signaling is essential for restoring TGFβ tumor suppressor function and could result in a paradigm shift for targeting TGFβ signaling to improve cancer therapy.

The administration sequence and mode of co-administration are key for the efficacy of combining TGFβ inhibitors with chemotherapeutic drugs. Most studies adopted a two-stage drug administration 34-38, 41, 44, 50, whereas other studies co-administrated the drugs 39, 40, 42, 45, 47, 48. Intriguingly, Pang et al. and Xia et al. corroborated that administration of TGFβ inhibitors before chemotherapy achieved better results than co-administration 36, 50. These studies also confirmed the significance of continuous administration of TGFβ inhibitors to enhance chemotherapy efficacy. Because TGFβ inhibitors were developed as oral medications for use in clinical settings, most studies used intragastric or intraperitoneal administration in tumor-bearing mice. However, intravenous administration of TGFβ inhibitors and chemotherapy drugs encapsulated in nanoparticles also inhibited tumor growth of both primary and metastatic tumors 33. Nonetheless, the optimal administration route remains to be determined in a clinical setting.

Which cancer patients will benefit from this combination therapy remains largely unexplored. Different cancer types have distinctive pathophysiological characteristics 172, which will require careful consideration before the administration of TGFβ inhibitors. It will likely not be feasible to target TGFβ signaling in all cancer type. For cancers with low expression of TGFβ ligands, TGFβ inhibition will have limited efficacy, and it may have adverse effects. Additionally, the efficacy of ECM remodeling and modulation of the tumor vasculature by targeting TGFβ signaling will also be determined by the pathophysiological characteristics of tumors. For hyperpermeable cancers with limited ECM and high vascularization 137, TGFβ inhibition may not be necessary to improve drug accumulation and penetration. However, patients with pancreatic, breast and hepatocellular carcinomas, which express high levels of TGFβ ligands, and have abundant tumor-associated ECM, and are poorly vascularized, might benefit significantly from treatment with TGFβ inhibitors. Indeed, a recent phase II clinical trial (NCT01821729) demonstrated that the combination of losartan and chemoradiotherapy provided clinical benefits for locally advanced pancreatic ductal adenocarcinoma 53. More clinical results are expected to emerge in the near future (Table S1).

The impact of combining TGFβ inhibitors with chemotherapy on the physical tumor microenvironment warrants further investigation. Studies have shown that mechanical forces had a significant influence on cancer progression and therapy 173-177, while the physical tumor microenvironment was largely determined by ECM contents 178, 179 and TGFβ signaling 180. Thus, TGFβ inhibition and subsequent ECM remodeling could effectively modulate the physical tumor microenvironment. This notion has been substantiated by several studies using tranilast 34-36, losartan 37-40, and PFD 41 in tumor-bearing mice. In addition, a retrospective analysis showed that losartan treatment significantly improved OS in women with ovarian cancer. Nevertheless, how the altered physical microenvironment affects tumor cells, and whether its modulation through TGFβ inhibition can increase the efficacy of anti-cancer therapies remains to be examined in depth in clinical settings.

Multiple TGFβ inhibitors are currently being tested in a clinical setting, with 6 ongoing phase III clinical trials (Table S1). The most promising candidates, M7824 and LY2157299, are currently being used in 13 and 18 clinical trials, respectively. In addition to TGFβ inhibitors, the FDA-approved drugs, losartan and PFD, are being tested in 9 and 5 clinical trials for cancer treatment, respectively. More clinical trial results are expected in the near future. Despite this progress, low therapeutic efficacy, serious adverse effects, and low patient recruitment have impeded the clinical translation of TGFβ inhibitors. Addressing these critical issues will promote the clinical translation of TGFβ inhibitors, which could enhance the efficacy of cancer chemotherapy.

Finally, targeting TGFβ signaling could be beneficial not only for chemotherapy but also for immunotherapy. TGFβ plays an essential role in creating an immunosuppressive microenvironment by polarizing M1 type macrophages to M2, N1 type neutrophils to N2, and by promoting naïve T cell differentiation to regulatory T cells 23, 24. Several studies have shown that TGFβ inhibition increased the antitumor efficacy of antibodies specific for PD-1, PD-L1, and CTLA-4 181-183. Our group showed that TGFβ inhibition increased the antitumor activity of commensal-derived probiotics 184. In clinical trials, bintrafusp alfa (M7824), a bifunctional fusion antibody of TGFβ/PD-L1 has used to treat biliary (NCT04066491, NCT03833661), non-small cell lung, and prostate cancers, recurrent respiratory papillomatosis, and other solid tumors (NCT03631706, NCT03493945, and NCT03707587). The TGFβ/PD-L1 antibody is also being tested in colorectal, pancreatic, small cell lung (NCT03436563, NCT03451773, and NCT03554473) and breast cancer (NCT03524170, NCT03620201) clinical trials. It is anticipated that these TGFβ targeting therapeutics will be applied in clinical settings in the near future.

## Supplementary Material

Supplementary table S1.

## Figures and Tables

**Table 1:** Targeting TGFβ pathway to enhance chemotherapy

Target	TGFβ inhibitors	Functions	Chemotherapeutics/ Nanotherapeutics	Synergistic effects	Cancer cell lines	Refs.
Cancer-associated fibroblasts	Tranilast	Reducing TGFβ1 expression; Attenuating pSmad2/3 levels and nuclear translocation	Doxil^®^, Abraxane^®^, DTX-Ms	Reducing collagen and hyaluronan levels. Alleviating solid stress and interstitial fluid pressure (IFP). Increasing the blood vessel perfusion. Enhancing drug delivery to tumors.	MCF10CA1a, 4T1, 3T3	33-35
	Pirfenidone	Reducing TGFβ1 expression; Attenuating pSmad2/3 levels	Doxorubicin	Reducing collagen and hyaluronan levels. Alleviating solid stress and IFP. Increasing blood vessel perfusion. Enhancing drug delivery to tumors.	MCF10CA1a, 4T1	40
	Losartan	Reducing TSP-1 expression and TGFβ1 activation	Doxorubicin, Doxil^®^, 5-FU, Paclitaxel, PTX-Cl-Lip	Reducing collagen and hyaluronan levels. Alleviating solid stress and IFP. Increasing blood vessel perfusion. Enhancing drug delivery to tumors.	E0771, AK4.4, FVB MMTV PyVT, L3.6pl, HSTS26T, SKOV3ip1, Hey-A8, 4T1	36-39
Pericytes	LY364947	Inhibiting TGFβ receptor I	DOX, Doxil^®^, DACHPt-loaded micelles, Gemcitabine-loaded liposomes	Decreasing vascular pericyte coverage. Enhancing nanoparticle extravasation from vasculature.	BxPC3, OCUM-2MLN	41-43
	1D11	Neutralizing TGFβ	Doxil^®^, Doxorubicin	Increasing vascular pericyte coverage. Normalizing tumor vasculature, and enhancing blood perfusion to increase drug delivery into tumors.	MDA-MB-231, 4T1	44
Cancer cells	LY2109761	Inhibiting TGFβ receptor I/II	Cisplatin	Inhibiting the growth and invasion of tumor cells.	MG-63	45
	LY2157299	Inhibiting TGFβ receptor I	Doxorubicin, DOX/LY@HES-PLA	Reversing EMT, overcoming drug resistance, and inhibiting both primary tumor growth and distant metastasis formation.	4T1	32
	Losartan	Reducing TSP-1 expression and TGFβ1 activation	PTX-loaded pH-sensitive cleavable liposomes	Suppressing tumor cell invasion and metastasis.	4T1	46
Cancer stem cells (CSCs)	LY2157299	Inhibiting TGFβ receptor I	Paclitaxel	Blocking CSCs expansion.	SUM159, BT549	47
	Silencing of YAP1 and IGF2BP3	Restoring the TGFβ signaling pathway	Rapamycin, Sorafenib	Inhibiting the oncogenic potential and chemoresistance of CSCs.	Huh7	48
	SB431542	Inhibiting ALK receptors	Cisplatin, Oxaliplatin, Doxorubicin	Inhibiting CSC stemness.	MHCC-97H, Huh7	49
