# Supplementary material for: Targeting transforming growth factor-β signaling for enhanced cancer chemotherapy
Source: Theranostics. 2021 Jan 1;11(3):1345–63. doi: 10.7150/thno.51383 (PMC7738904; doi:10.7150/thno.51383)
Supplement: Supplementary file 1 — Supplementary table S1. [file thnov11p1345s1.pdf]

Table S1. Summary of TGFβ Targeting Drugs in Pre-clinical Studies and Clinical Trials for Different Cancers

Drug	Cancer type	Targets	Phases	NCT Number	Status
Antisense oligonucleotides					
Trabedersen (AP12009)	Pancreatic neoplasms, melanoma, colorectal neoplasms	TβRII	Phase I	NCT00844064	Completed
	Glioblastoma, anaplastic astrocytoma	TβRII	Phase II	NCT00431561	Completed
	Anaplastic astrocytoma, glioblastoma	TβRII	Phase III	NCT00761280	Terminated, has results
Lucanix	Lung neoplasm, bronchogenic carcinoma	TβRII	Phase II	NCT01058785	Completed
	Lung cancer	TβRII	Phase III	NCT00676507	Completed
Vigil	Ewings sarcoma, non small cell lung cancer, liver cancer	TGFβ1+TGFβ2	Phase I	NCT01061840	Completed
	Breast cancer, ovarian cancer, fallopian tube cancer, primary peritoneal carcinoma, uterine cancer, cervical cancer, endometrial cancer	TGFβ1+TGFβ2	Phase II	NCT02725489	Active, not recruiting
	Advanced gynecological cancers, ovarian cancer, cervical cancer, uterine cancer	TGFβ1+TGFβ2	Phase II	NCT03073525	Active, not recruiting
	Colon cancer	TGFβ1+TGFβ2	Phase II	NCT01505166	Terminated, has results
	Advanced melanoma	TGFβ1+TGFβ2	Phase II	NCT01453361	Terminated, has results
	Ovarian cancer	TGFβ1+TGFβ2	Phase II	NCT01309230	Active, not recruiting
	Ovarian cancer, ovarian neoplasms	TGFβ1+TGFβ2	Phase II	NCT02346747	Active, not recruiting
	Ovarian cancer	TGFβ1+TGFβ2	Phase II	NCT01867086	Completed, has results
	Ovarian cancer	TGFβ1+TGFβ2	Phase II	NCT01551745	Completed, has results
	Ewing's sarcoma	TGFβ1+TGFβ2	Phase III	NCT03495921	Active, not recruiting
	Ewing sarcoma	TGFβ1+TGFβ2	Phase II	NCT02511132	Active, not recruiting
	Recurrent melanoma, malignant melanoma	TGFβ1+TGFβ2	Phase I	NCT02574533	Completed
Antibodies					
Fresolimumab (GC-1008)	Non-small cell lung carcinoma	TβRI+TβRII	Phase I/II	NCT02581787	Recruiting
	Primary brain tumors	TβRI+TβRII	Phase II	NCT01472731	Completed
	Metastatic breast cancer	TβRI+TβRII	Phase II	NCT01401062	Completed, has results
	Renal cell carcinoma	TβRI+TβRII	Phase I	NCT00923169	Completed
	Renal cell carcinoma, melanoma	TβRI+TβRII	Phase I	NCT00356460	Completed
	Pleural malignant mesothelioma	TβRI+TβRII	Phase II	NCT01112293	Completed, has results
PF-03446962	Transitional cell carcinoma of bladder	TβR ALK1	Phase II	NCT01620970	Unknown status
	Advanced solid tumors	TβR ALK1	Phase I	NCT00557856	Completed, has results
	Neoplasms	TβR ALK1	Phase I	NCT01337050	Completed, has results

	Hepatocellular carcinoma	TβR ALK1	Phase II	NCT01911273	Terminated, has results
	Colorectal cancer	TβR ALK1	Phase I	NCT02116894	Completed
	Malignant pleural mesothelioma	TβR ALK1	Phase II	NCT01486368	Completed
Bintrafusp Alfa (M7824)	Urothelial cancer, bladder cancer	TGFβ	Phase I	NCT04235777	Not yet recruiting
	Head and neck Cancer	TGFβ	Phase I/II	NCT04247282	Not yet recruiting
	Breast cancer	TGFβ	Phase I	NCT03620201	Recruiting
	Non-small cell lung cancer	TGFβ	Phase I/II	NCT04297748	Not yet recruiting
	Lung cancer	TGFβ	Phase I/II	NCT03554473	Recruiting
	Breast cancer	TGFβ	Phase I	NCT03524170	Recruiting
	Breast cancer	TGFβ	Phase I	NCT04296942	Not yet recruiting
	Breast cancer	TGFβ	Phase I	NCT03579472	Recruiting
	Colon cancer, rectal cancer	TGFβ	Phase I/II	NCT03436563	Recruiting
	Uterine cervical neoplasms	TGFβ	Phase II	NCT04246489	Not yet recruiting
	Biliary tract cancer, cholangiocarcinoma, gallbladder cancer	TGFβ	Phase II	NCT03833661	Recruiting
	Biliary tract cancer, cholangiocarcinoma, gallbladder cancer	TGFβ	Phase II/III	NCT04066491	Recruiting
	Recurrent head and neck squamous cell carcinoma	TGFβ	Phase I/II	NCT04220775	Not yet recruiting
GS-1423	Advanced solid tumors	TGFβ	Phase I	NCT03954704	Recruiting
NIS793	Breast cancer, lung cancer, hepatocellular cancer, colorectal cancer, pancreatic cancer, renal cancer	TGFβ	Phase I	NCT02947165	Recruiting
1D11	Breast cancer	TGFβ	Preclinical		
TβR kinase inhibitors					
Galunisertib (LY2157299)	Prostate cancer	TβRI	Phase II	NCT02452008	Recruiting
	Metastatic breast cancer	TβRI	Phase II	NCT02538471	Terminated, has results
	Rectal adenocarcinoma	TβRI	Phase II	NCT02688712	Recruiting
	Recurrent non-small cell lung cancer, recurrent hepatocellular carcinoma	TβRI	Phase I/II	NCT02423343	Active, not recruiting
	Carcinosarcoma, ovarian	TβRI	Phase I	NCT03206177	Recruiting
	Hepatocellular carcinoma	TβRI	Phase II	NCT02178358	Active, not recruiting
	Glioma	TβRI	Phase I/II	NCT01220271	Completed
	Advanced hepatocellular carcinoma (HCC)	TβRI	Phase I	NCT02906397	Active, not recruiting
	Metastatic pancreatic cancer	TβRI	Phase I	NCT02734160	Completed
	Pancreatic neoplasms	TβRI	Phase I	NCT02154646	Completed
	Neoplasms, neoplasm metastasis, pancreatic cancer	TβRI	Phase I/II	NCT01373164	Completed, has results

	Neoplasms, neoplasm metastasis	TβRI	Phase I	NCT01722825	Completed
	Neoplasm	TβRI	Phase I	NCT02304419	Completed
	Hepatocellular carcinoma	TβRI	Phase I	NCT02240433	Completed
	Colorectal cancer metastatic	TβRI	Phase I/II	NCT04031872	Not yet recruiting
	Carcinoma, hepatocellular	TβRI	Phase II	NCT01246986	Active, not recruiting
	Glioblastoma	TβRI	Phase II	NCT01582269	Active, not recruiting
	Glioma	TβRI	Phase I	NCT01682187	Active, not recruiting
TEW-7197	Advanced stage solid tumors	TβRI	Phase I	NCT02160106	Completed
	Metastatic pancreatic cancer	TβRI	Phase I/II	NCT03666832	Recruiting
	Metastatic non-small cell lung cancer	TβRI	Phase I/II	NCT03732274	Recruiting
	Urothelial carcinoma, advanced urothelial carcinoma	TβRI	Phase II	NCT04064190	Not yet recruiting
	Multiple myeloma	TβRI	Phase I	NCT03143985	Recruiting
	Metastatic gastric cancer	TβRI	Phase I	NCT03698825	Recruiting
	Metastatic colorectal cancer, gastric cancer	TβRI	Phase I/II	NCT03724851	Recruiting
	Pancreas cancer	TβRI	Phase I	NCT04258072	Not yet recruiting
	Myeloproliferative neoplasm	TβRI	Phase II	NCT04103645	Active, not recruiting
LY3200882	Colorectal cancer metastatic	TβRI	Phase I/II	NCT04031872	Not yet recruiting
	Solid tumor	TβRI	Phase I	NCT02937272	Active, not recruiting
LY364947	Pancreatic cancer, scirrhous gastric cancer	TβRI	Preclinical		
LY2109761	Osteosarcoma	TβRI+TβRII	Preclinical		
SB431542	Hepatocellular carcinoma	ALK Receptor	Preclinical		
TGFβ traps					
AVID200	Malignant solid tumor	TGFβ	Phase I	NCT03834662	Recruiting
	Myelofibrosis	TGFβ	Phase I	NCT03895112	Recruiting
Other small-molecule inhibitors					
Losartan	Pancreatic cancer	TGFβ1	Phase II	NCT03563248	Recruiting
	Pancreatic cancer	TGFβ1	Phase II	NCT01821729	Active, not recruiting, has results
	Pancreatic cancer	TGFβ1	Phase I	NCT04106856	Recruiting
	Lung cancer, atrial fibrillation	TGFβ1	Phase III	NCT01281787	Completed
	Pancreatic cancer	TGFβ1	Early Phase I	NCT01276613	Terminated
	Advanced solid tumors, metastatic melanoma	TGFβ1	Phase I	NCT03864042	Recruiting
	Glioblastoma	TGFβ1	Phase II	NCT03951142	Recruiting

	Osteosarcoma	TGFβ1	Phase I	NCT03900793	Recruiting
	Newly-diagnosed glioblastoma	TGFβ1	Phase III	NCT01805453	Unknown status
Pirfenidone	Lung cancer	TGFβ	Phase I	NCT03177291	Recruiting
	Neurofibromatosis, plexiform	TGFβ	Phase II	NCT00076102	Completed, has results
	Neurofibromatosis	TGFβ	Phase II	NCT00754780	Completed
	Neurofibromatosis, precancerous condition	TGFβ	Phase I	NCT00053937	Completed
	Uterine leiomyoma, fibroids	TGFβ	Phase II	NCT00332033	Completed
Tranilast	Breast cancer	TGFβ+Smad2/3	Preclinical		
